# The Significance of Targeting Poly (ADP-Ribose) Polymerase-1 in Pancreatic Cancer for Providing a New Therapeutic Paradigm

**DOI:** 10.3390/ijms22073509

**Published:** 2021-03-29

**Authors:** Keun-Yeong Jeong, Min Hee Park

**Affiliations:** Research Center, MetiMedi Pharmaceuticals Co., Incheon 22006, Korea; pmh1880@hanmail.net

**Keywords:** pancreatic cancer, PARP-1, oxidative stress, mitosis, genome instability, transcription, PARP-1 inhibitor

## Abstract

Genome-wide studies focusing on elucidating the effects on cancer progression have enabled the consequent identification of a distinct subpopulation of pancreatic cancer cells with unstable genomic characteristics. Based on this background, deleterious changes by poly (adenosine diphosphate (ADP)-ribose) polymerase-1 (PARP)-1 have been concentrated in oncology. One of the critical functions of PARP-1 is the response to DNA damage, which plays a pivotal role in DNA repair in cancers. PARP-1 also has widespread functions that are essential for the survival and growth of cancer cells. It regulates oxidative stress in mitochondria through the regulation of superoxide and oxidation. PARP-1 is in charge of regulating mitosis, which is a crucial role in tumorigenesis and remodels histones and chromatin enzymes related to transcriptional regulation, causing alterations in epigenetic markers and chromatin structure. Given the significance of these processes, it can be understood that these processes in cancer cells are at the frontline of the pathogenetic changes required for cancer cell survival, and these contributions can result in malignant transformation. Therefore, this review addresses the current molecular biological features for understanding the multifactorial function of PARP-1 in pancreatic cancer related to the aforementioned roles, along with the summary of recent approaches with PARP-1 inhibition in clinical studies targeting pancreatic cancer. This understanding could help to embrace the importance of targeting PARP-1 in the treatment of pancreatic cancer, which may present the potential to find out a variety of research topics that can be both challenged clinically and non-clinically.

## 1. Introduction

Unfortunately, pancreatic cancer is typically diagnosed at an advanced stage due to the difficulty of recognizing the symptoms at an early stage. The 5-year survival rate is <9%, and 74% of all patients with pancreatic cancer die within the first year of diagnosis [1]. Therefore, there exists an important challenge of widening the spectrum of anticancer drugs targeting pancreatic cancer to achieve clinical benefits and improvement of symptoms even in an advanced stage. To date, only a few options for the treatment of pancreatic cancer have been suggested [2]. Use of gemcitabine alone or in combination with existing anticancer drugs such as capecitabine and a combination of fluorouracil, leucovorin, irinotecan, and oxaliplatin (FOLFIRINOX), or nap-paclitaxel is the recent choice for treatment [2]. Although a trend toward the improved duration of survival in patients who received FOLFIRINOX or nab-paclitaxel-gemcitabine protocols has been demonstrated in comparison with fixed-dose gemcitabine plus capecitabine, it could not incorporate all the unexpected features of the genetic alterations in pancreatic cancer [3,4]. Moreover, these regimens come at the expense of increased toxicity with high rates of leukopenia, diarrhea, polyneuropathy, and infectious complications following an incremental activity of recent regimens [5,6]. Genetic alterations are characterized by mutations in oncogenes; typically, the majority of pancreatic cancers harboring drug resistance are related to kirsten rat sarcoma viral oncogenes homolog (KRAS) mutation among about 90% of the patients [7]. Therefore, the strategy of targeting KRAS to treat pancreatic cancer has been applied at different stages of KRAS molecular intracellular processes, such as anti-KRAS peptides and downstream signaling inhibitors [8,9]. However, despite some encouraging results in the non-clinical stage, no noteworthy clinical benefits have been gained yet. Hence, another perspective of the molecular mechanisms underlying the pathogenesis of pancreatic cancer for overcoming their chemoresistance will be required to develop novel strategies to provide effective care.

**Figure 1:**
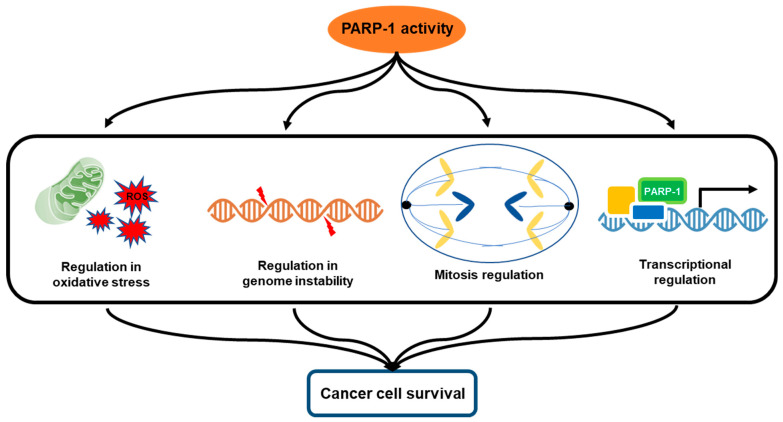
Multifactorial role of poly (adenosine diphosphate (ADP)-ribose) polymerase-1 (PARP-1) in cancer cell survival. PARP-1 can occupy a position as an important regulator of mitochondrial superoxide and oxidation, and it is required for the dysfunctional regulation of the cell cycle and cell proliferation that has a crucial role in tumorigenesis. Moreover, PARP-1 can modulate histone proteins, epigenetic markers, and chromatin structure related to transcriptional regulation and DNA repair under genome instability. Therefore, PARP-1 has critical relevance to the development and malignant transformation of pancreatic cancer.

Since the primary reason for the onset of pancreatic carcinogenesis is genome instability, recent studies have elucidated the effects on cancer progression, particularly related to DNA damage response [10]. Genome-wide studies have allowed the consequent identification of a distinct subpopulation of pancreatic cancer cells with unstable genomic characteristics due to mutations in DNA repair genes [10]. Based on this background, there has been a focus on a high frequency of deleterious changes within the genomic structure as a consequence of impaired DNA repair response. Poly (adenosine diphosphate (ADP)-ribose) polymerase-1 (PARP-1) is an essential nuclear enzyme of cellular homeostasis that modifies several nuclear proteins by poly ADP-ribosylation. One of the important functions of PARP-1 is inducing the response to DNA damage, and the upregulation of PARP-1 in cancer along with its pivotal role in DNA repair has led to the investigation of the targetability of this critical enzyme [11]. Furthermore, PARP-1 has widespread functions that are vital for essential cellular homeostasis, and the following roles in the microenvironment of tumors are drawing attention [12]. PARP-1 regulates mitochondrial activity by occupying a prominent position that is characterized by the regulation of mitochondrial superoxide and oxidation [13]. It is required for the dysfunctional regulation of the cell cycle and cell proliferation that has a crucial role in tumorigenesis [13]. Moreover, upon PARP-1 activation, modulation of epigenetic markers and chromatin structure occurs by remodeling histones and chromatin enzymes through direct and indirect pathways related to transcriptional regulation [13,14]. Considering the significance of the abovementioned processes, it is understandable that PARP-1 has critical relevance during cancer development. These cellular processes contributed by PARP-1 can result in malignant transformation, and the cancer cells would be more likely to adapt better even under unfavorable conditions to survival (Figure 1). Therefore, the approach of targeting PARP-1 is emerging as a promising therapeutic strategy for targeting the pathogenesis in pancreatic cancer. This review summarizes the current molecular biological knowledge to understand the multifactorial functions of PARP-1 that enable the progression of pancreatic cancer, and the approaches and outcomes of PARP-1 inhibition in clinical studies targeting pancreatic cancer are also discussed.

## 2. PARP-1 and Poly-ADP Ribosylation

**Figure 2:**
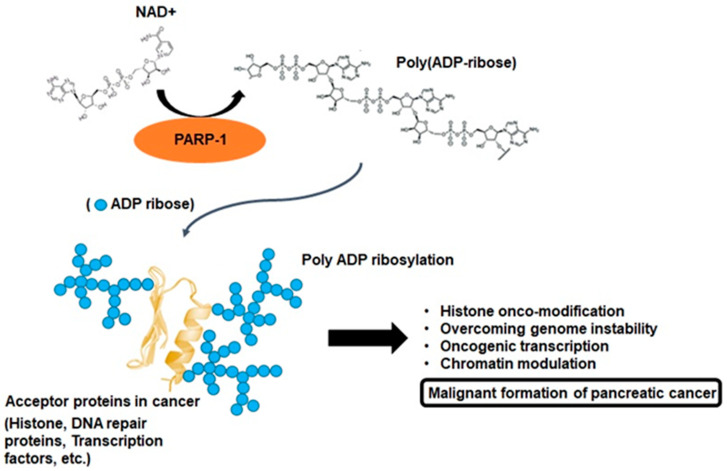
Poly ADP-ribosylation in cancer. PARP-1 branched poly ADP-ribose polymers following the cleavage of nicotinamide adenine dinucleotide (NAD+) to ADP-ribose. PARP-1 enables interactions by catalyzing the covalent attachment of poly ADP-ribose polymers on acceptor proteins, such as histones, DNA repair proteins, transcription factors, and chromatin modulators. This enzymology reaction is known as poly ADP-ribosylation, and this process may be important for pancreatic cancer malignant formation.

To interpret the various cellular physiological functions of PARP-1, it is extremely important to understand the biochemical phenomenon defined as poly-ADP-ribosylation (PARylation). PARP-1 consists of a multidomain structure sharing the same catalytic domain that exhibits structural homology to other ADP-ribosyl transferases [15]. The N-terminal region contains a DNA-binding domain with three zinc fingers and an auto-modification domain. The C-terminal region contains the protein-interacting domain and the catalytic subdomain responsible for ADP-ribosylation reaction [15]. These domains enable genetic interactions by catalyzing the covalent attachment of poly-ADP-ribose (PAR) polymers on PARP-1 and other acceptor proteins, including histones, DNA repair proteins, transcription factors, and chromatin modulators [15,16]. PARP-1 and tankyrases synthesize branched PAR polymers following the cleavage of nicotinamide adenine dinucleotide to nicotinamide and ADP-ribose, and this enzymology reaction is the critical process for PARylation (Figure 2) [16]. These resemble the grab hold of PARP-1 to DNA transcription factors, protein–protein, and protein–nucleic acid interactions [12,16]. Although the majority of cellular PARP-1 activity is localized to the nucleus, PAR and PARylated proteins can also be transferred to the cytosol. Therefore, the molecular biological aspects of PARP-1 activity can exert various roles through the pathophysiological outcomes with PARylation [11,13,16,17]. This rationale can also be accounted for the feature of a unique enzymatic event, namely, PARP-1 activation, with PARylation-mediated cellular physiological changes being associated with the development of cancer [16,17]. This process is characterized by alterations at the cellular, genetic, and epigenetic levels comprising a multistep process involving various physiological maintenance and overcoming stress conditions [13,16,17]. PARP-1 and PARylation have been implicated in all these processes, suggesting possible connections between PARP-1 function and tumorigenesis [16,17]. In other words, a series of biochemical phenomena associated with PARylation following PARP-1 activation can be a potential theory that can collectively explain the multifactorial process of pancreatic cancer progression (Figure 2).

## 3. Multifactorial Functions of PARP-1 in Cancer Progression

### 3.1. Oxidative Stress and PARP-1

Oxidative phosphorylation is the basic metabolism responsible for the process of generating energy in the mitochondria, and as a result of this metabolism, essential compounds are also synthesized that are beneficial for the cell to survive [18]. However, the result of this metabolism also causes the cells to undergo oxidative stress due to the production of reactive species following the activation of mitochondrial enzymes [19]. Reactive oxygen species (ROS) account for a high proportion among these reactive species within the cells, and low concentration levels (<5%) of these species can be involved in benign metabolic events such as cell signaling, enzyme activation, and gene expression [19,20]. However, the loss of equilibrium between ROS and endogenous antioxidant species induces intracellular oxidative stress and alteration and damage to several intracellular molecules, including DNA, RNA, lipids, and proteins, even leading to the progression of apoptosis [19]. Nevertheless, cancer cells can rather function positively under higher levels of oxidative stress conditions, which indicates the possibility that unique adaptive responses to oxidative stress would be an essential factor contributing to malignant transformation, including DNA damage, cell–cell adhesion, and signaling for sustained cancer cell survival [20]. Regulating the balance or imbalance of intracellular ROS to maintain cellular functions is a well-established hallmark of pancreatic cancer cells. Maintaining a moderately higher level of ROS production than that in normal cells in the tumor microenvironment can act as a signaling molecule to promote the mutation of genomic DNA or enhance the proliferation of pancreatic cancer cells [21]. On the one hand, excess ROS can cause irreversible oxidative damage, induce cell death through apoptosis, necrosis, and autophagy, and render pancreatic cancer cells susceptible to extracellular turbulences such as chemotherapy and radiation therapy [20,21]. Under such a critical environment, the functioning of the antioxidant program protects the pancreatic cancer cells from irreversible oxidative damage. The antioxidant program is driven by defense through enzymatic antioxidants, including the detoxification of secondary metabolites, as well as the direct removal of the electrophile itself [22]. Interestingly, PARP-1 controls a core role in the areas of such active utilization of oxidative stress conditions similar to a double-edged sword.

**Figure 3:**
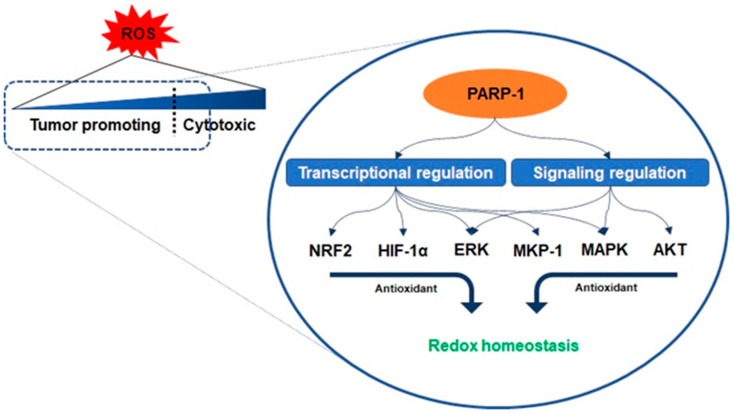
Regulation of redox homeostasis by PARP-1. The activity of PARP-1 plays an important role in the adaptive response of cancer cells to oxidative stress. PARP-1 promotes the interaction of antioxidant response elements with nuclear factor erythroid-related factor 2 (NRF2) and interacts with hypoxia-inducing factor 1-alpha (HIF1-α). PARP-1 activation and poly ADP ribose synthesis possibly modulate the mTOR complex 2, resulting in AKT downregulation to inhibit oxidants’ accumulation. To regulate oxidants level, PARP-1 is in charge of the transcription of the extracellular signal-regulated kinase (ERK) and mitogen-activated protein kinase phosphatase 1 (MKP-1) related to mitogen-activated protein kinase (MAPK) regulation.

Antioxidant enzymes are under the transcriptional control of nuclear factor erythroid-related factor 2 (NRF2), a basic leucine zipper protein, and respond to physiological changes between intracellular redox actions for maintaining cellular homeostasis [23]. Because PARP-1 promotes the interaction of antioxidant response elements with NRF2 and NRF2-partners, their interaction with PARP-1 enables the entire transcriptional activity of NRF2 [24]. Another counteracting mechanism responsible for oxidant-involving PARP-1 is denoted by its interaction with hypoxia-inducing factor 1-alpha (HIF1-α), which undergoes activation during hypoxia-triggered redox imbalance [25,26]. PARP-1 forms a co-transcriptional activator with HIF1-α and enables the expression of the genes required for the maintenance of glutathione homeostasis, such as heme oxygenase-1 and glucose transporter 1, controlled in a PARP-1-dependent manner in a hypoxic response element promoter region [25]. Furthermore, recent studies have reported that the signaling on ROS is dependent on PARP-1 activation related to the regulation of protein kinase B (AKT) [27,28,29]. Cancer cells with KRAS mutation display constitutive activation of the AKT pathways, resulting in ROS production [29]. As mentioned above, low concentration levels of ROS can be involved in benign metabolic events, but excessive ROS induces intracellular oxidative stress, such as damage to several intracellular molecules, leading to apoptosis [19,20]. The cascade in the AKT pathway, phosphatidylinositol 3, acts as a redox sensor and phosphorylates AKT to induce the active form, and AKT stimulates oxidative metabolism and forkhead box O-dependent catalase inhibition, contributing to the accumulation of hydrogen peroxide, however, PARP-1 activation and PAR synthesis inhibit the mTOR complex 1 signaling pathway and possibly modulate the mTOR complex 2, resulting in AKT downregulation [27,30,31]. The increase in the levels of oxidants enhances the expression of the extracellular signal-regulated kinase (ERK) followed by the regulation of oxidant levels in the cells [27]. PARP-1 involves the transcription of ERK and mitogen-activated protein kinase phosphatase 1 (MKP-1) in these signaling events, thereby regulating mitogen-activated protein kinase (MAPK) [32]. Its regulation for the downregulation of MKP-1 blocks the dephosphorylation of the tyrosine and threonine residues of MAPK, which are activated upon acute oxidant exposure [32]. In other words, PARP-1 is involved in blocking the dephosphorylation of the tyrosine and threonine residues in MAPK, thus contributing to the induction of sustained survival signals even under the increase in ROS (Figure 3) [25,32].

### 3.2. Genomic Instability and PARP-1

Maintenance of genomic stability is important for cellular integrity in normal cells to prevent mutation by endogenous genotoxic stresses or exogenous carcinogenic insults. In contrast, carcinogenesis progresses under genomic alterations in cells accompanied by the selection of aggressive subclones in this process. These conditions provide growth benefits to cancer cells under a genetically unstable state and result in select malignant formation [33]. Tumor progression requires an increase in genetic alterations and downregulation of DNA damage surveillance mechanisms to achieve uncontrolled proliferation for aggressive growth [34]. The insights provided through studies on oncogenes could indicate that long-lasting stress from excessive replication promotes genomic instability with sustained damage to DNA [33,34]. In pancreatic cancer, silencing or detrimental mutations in the key gene responsible for DNA damage response or tumor suppressor genes, such as breast cancer susceptibility gene (BRCA), ataxia telangiectasia mutated (ATM), and p53, have been associated with increased genomic instability [35]. BRCA mutations are exposed susceptible to intrinsic or extrinsic stress because of the lack of DNA repair response, and a mutation in the ATM gene causes increased genetic alterations such as deletion or insertion of new nucleotides and chromosomal translocations [34,35]. The major tumor suppressor gene p53 can contribute to high genomic instability following the loss of activity [35]. In other words, the result of genomic instability is a continuing genetic rearrangement throughout the incidence and progression of pancreatic cancer, and various genetic changes have been observed at different loci under metastatic characteristics [33,34,35]. This indicates the possibility that they possess a variety of cancer properties with resistance, even in the same metastatic lesions from the same parental clone.

**Figure 4:**
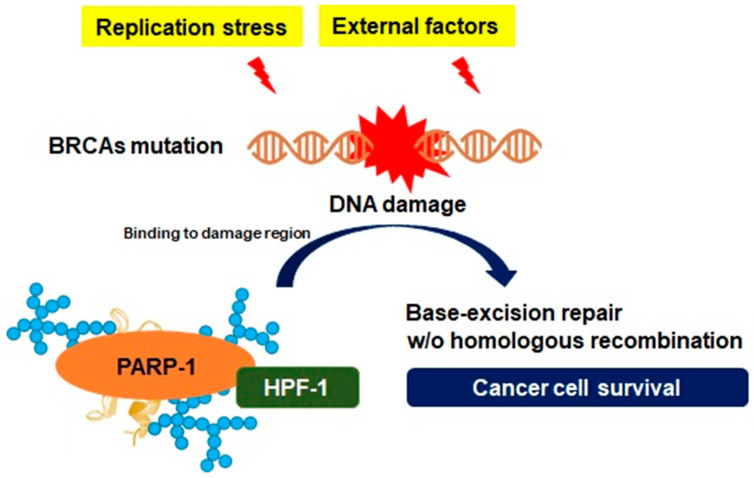
Adaptation of genomic instability depending on PARP-1. Silencing or detrimental mutations in the DNA damage response genes, such as the breast cancer susceptibility gene (BRCA), are associated with genomic instability to achieve uncontrolled proliferation for aggressive growth in cancer cells. However, such an environment could be an unfavorable condition for cancer cell survival, where persistent DNA damage is induced. However, PARP-1 can facilitate DNA repair in response to replication stress or external factors by maintaining the open form of chromatin structures following PAR synthesis on acceptor proteins, such as histone PARylation factor 1 (HPF-1), which is available to the adaptation to genomic instability.

Genetic instability is generated by excessive replication stress, and either directly or indirectly, it leads to DNA damage initiated by single-stranded DNA (ssDNA) gaps or double-stranded DNA (dsDNA) breaks, followed by the generation of the DNA repair system [36]. Homologous recombination is a DNA metabolic process found in most types of cells, providing a form of repair targeting complex DNA damage, cross-linking between ssDNA gap and dsDNA break [37]. DNA damage response gene-mutated pancreatic cancer cells drive a unique DNA repair system that creates specific genotypic and phenotypic features known as BRCAness. The patterns of BRCAness can provide genetic plasticity, and a variety of molecular behaviors and such genotypes are involved in the process of constructive genomic rearrangement, which can directly alter the genomic structure and molecular properties of pancreatic cancer cells [38]. Another feature that is not directly involved in the DNA repair process of homologous recombination in pancreatic cancer is PARP-1 activation [39]. As described in the previous section, PARP-1 is a crucial nuclear enzyme of cellular homeostasis that modifies several nuclear proteins by PARylation [16]. One of the key features of PARP-1 is to repair ssDNA breaks in response to DNA damage by targeting histone core and the linker histone proteins in the nucleus [16]. Although the detailed roles of histone modifications with PARP-1 need to be elucidated, previous studies have suggested that PARP-1 can facilitate DNA repair by maintaining the open form of chromatin structures [40]. A specific serine group-bound ADP-ribose depends on a protein known as histone PARylation factor 1, which has been identified as a key protein controlling the DNA damage-induced PARylation and is available to the adaptation to genomic instability [41]. As PARP-1 constantly recruits elements for DNA repair via PARylation on multiple receptor regions under genomic instability, histone PARylation factor 1 also has an essential role in the regulation of excessive PARP-1 transformation to evade cell death by apoptosis-inducing factors [12]. In other words, since the abnormal growth of pancreatic cancer constantly induces DNA damage, leading to genomic instability, the PARP-1 activity and PARylation play a major role in the adaptation to genomic instability in pancreatic cancer (Figure 4).

### 3.3. Mitosis Regulation

Cell division in eukaryotes is a biological process required for the generation of progeny cells, and somatic cell proliferation is controlled via mitosis [42]. Mitosis comprises four phases within a large frame. Prophase involves mobilization of nuclear fission, chromosomal condensation, centrosome separation, and recruitment of mitotic checkpoint proteins. The cell division then follows metaphase and anaphase and ends with telophase, which completes the processes of chromosomal atrophy and nuclear envelope reassembly around polar chromosomes [42]. All these processes comprise a sequence to efficiently eliminate errors during mitosis, and accuracy and efficiency are maintained by appropriate regulation of the expression and function of mitotic proteins. This activity is regulated by the mechanisms of phosphorylation and ubiquitination associated with post-translational modification of the gene [42,43]. However, the occurrence of a defect can induce abnormal mitosis linking with genetic instability, which can be considered as a hallmark of cancer formation [44]. The major regulatory function of mitosis depends on the balanced level of the spindle assembly checkpoint protein. Modified expression of the spindle assembly checkpoint protein has been reported in a variety of cancers, and these defects often rely on aneuploidy in normal cells [45]. Furthermore, defects in chromosome segregation during mitosis can lead to aneuploidy and contribute to genomic instability [46]. Recent studies have identified mutations for modulators that are important in encoding the protein subunits of the segregation complex in many aneuploidy cancer cells [47]. Defects in several of these molecular components or upstream regulation of irregulated chromosome numbers and cellular proteins that control poly-polarity, such as the checkpoint kinase and cyclin family proteins, can upregulate the proliferation of cancer cells [47].

**Figure 5:**
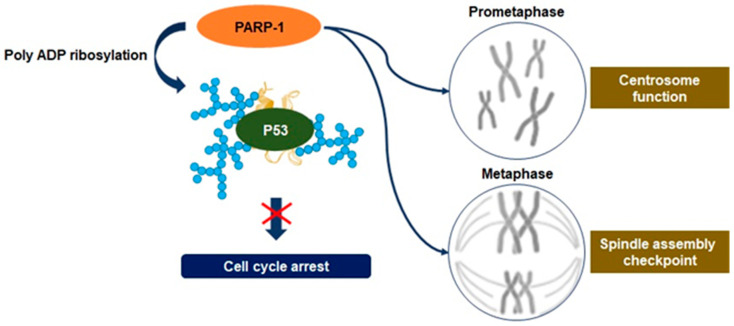
Regulation of mitosis by PARP-1. Defects in chromosome segregation during mitosis can lead to many aneuploidies, and upstream regulation of irregulated chromosome numbers can upregulate the proliferation of cancer cells. PARP-1 regulates the function of the centrosome to control spindle assembly checkpoints in cancer cells, and it may result from excessive poly ADP ribosylation on the tumor suppressor gene p53. PARP-1 is accumulated in centrosome chromatin during mitotic metaphase and can modify spindle assembly checkpoints, leading to aneuploidy. These activities have a role in promoting tumorigenesis of pancreatic cancer cells via aneuploidy-induced delaminating properties.

Recent studies have disclosed an association between the upregulation of PARP-1 activity and mitosis. It has been observed that PARP-1 activity regulates the function of the centrosome to control anaphase and spindle assembly checkpoints [48,49]. Moreover, PARylation of mitotic chromatin can contribute toward serving as an indicator of epigenetic regulation at the site of transcription initiation required for transcription reactivation following mitosis [14,48]. The centrosome is the primary site of microtubule nucleation required for the formation of new microtubules through the assembly of the mitotic spindle. Therefore, centrosome dysfunction impairs chromosome segregation, promoting aneuploidy and inducing cancerous transformation [50]. Although the substrate of PARP-1 involved in the regulation of centrosome function has not been characterized, the association between PARP-1 and the centrosome throughout the cell cycle has been inferred by research via PARP-1 inhibition [51]. It may result from excessive PARylation on the tumor suppressor gene p53, one of the PARP-1 substrates known to regulate centrosome redundancy [51]. PARP-1 is also accumulated in centrosome chromatin during mitotic metaphase and is dissociated in anaphase following the interaction with centrosome proteins (Figure 5) [48]. Therefore, depletion of PARP-1 causes incomplete synapsis of homologous chromosomes and deficient sister chromatid cohesion [48,52]. PARP-1 activity can modify spindle assembly checkpoints through the degradation of cyclin B1 and the downregulation of cyclin-dependent 1 kinase activity, highlighting the point where excessive levels of PARylation have a critical role in the regulation of spindle assembly checkpoints, leading to aneuploidy [53]. These activities enable driving the tumorigenesis of pancreatic cancer cells via aneuploidy-induced delaminating properties and represent the characteristic of overcoming replication stress [47,48].

### 3.4. Transcriptional Regulation

As genetic dysregulation is one of the primary features of cancer, it is important to gain insights into the specific transcription process involved in the pathogenesis in the cancers. Cutting-edge genomic sequencing conducted to date has allowed us to identify key mutations that affect the transcriptional components and also made it possible to help understand cancer progression by transcriptional regulation. The majority of cancer cells have a feature of constantly maintaining a tumor microenvironment that is favorable for survival through the modification of gene regulators or mutations in signaling factors that are converged on transcriptional regulations [54]. Genetic variations in cancer can be influenced by changes in proteins that participate in any stage of transcriptional regulation, including transcription factors, co-factors, and promoters [55]. The final result of these alterations, especially the most profound issues with gene expression, is to contribute to the formation of malignancies [54,55].

Pancreatic cancer can be influenced by a variety of transcriptional regulations, and its activation or inactivation by mutations in transcription factors contributes to this function. The members of the microphthalmia-related transcription factor, transcription factors E3 and EB, regulate the expression of genes associated with high lysosomal activity required for the growth of pancreatic cancer [56]. Pancreatic duodenum homeobox protein 1 plays a vital role in regulating the early stages of pancreatic development. The pancreas transcription factor 1 subunit-alpha is an essential element that plays an essential role in pancreatic cancer cell differentiation for the early development of pancreatic cancer [57]. Nuclear receptor subfamily 5 group A member 2, known as liver receptor homolog 1, is a key molecule for the development of pancreatic cancer and is a direct downstream target of pancreatic duodenum homeobox protein 1 [58]. Hepatocyte nuclear factors, also known as transcription factor 1, belong to the homeobox family of proteins that play vital roles in the development of beta cells during pancreatic tissue formation [59]. All these transcription factors can create a network for interactions that regulate the development of pancreatic cancer. However, before referring to the action of such transcription factors, histone modifications or chromatin remodeling should be prioritized and interactions with enzymes such as the nucleosome can be emphasized essentially. Obviously, PARP-1 not only enables the interaction with nucleosome- and chromatin-related proteins, including transcription factors and components, but it also enables direct communication with DNA [24,60]. Therefore, it can be stated that PARP-1 can also be considered as an important marker for identifying the critical relationship between transcription regulation and the development of pancreatic cancer.

PARP-1 is a DNA-dependent ADP-ribosyl transferase that is confined to the nucleus and is frequently associated with chromatin [14,40]. The role of PARP-1 activity is not only bound to damaged DNA or other nuclear proteins, but it also includes post-translational modifications by PARylation [16,17,40]. Therefore, it has been demonstrated that PARP-1 accomplishes multiple roles in transcriptional regulation in cancer cells, including properties closely related to genome maintenance, such as DNA repair [24,40]. PARP-1 can alter the structure of nucleosomes and chromatin to a form that affects chromatin structure by binding to nucleosomes, compressing chromatin, and inducing PARylation in histones [14,40]. Moreover, PARP-1 is localized to the promoter of the transcribed genes and promotes transcriptional activity by interrupting histone binding. Because the transcriptional regulation function of PARP-1 does not generally require co-activity with other enzymes, various functions such as enhancer-binding, regulation of chromatin structure, and transcription factor regulation can independently progress [61]. The binding of PARP-1 can consistently demonstrate transcriptional regulation, positively or negatively, through several mechanisms, and appear differently according to the demands of the tumor microenvironment (mostly favorable directions for survival). PARP-1 can also immediately regulate the sequence-specific transcription factors that are highly relevant for malignant tumor formation. It constitutes a transcriptional inhibitory complex with p53, and in this context, PARylation can result in the mobilization of histone deacetylases for p53 suppression, leading to increased expression of oncogenes associated with disease progression and metastasis [62]. PARP-1-dependent interruption of metastasis-associated protein 1 gene expression results in enhanced levels of hypoxia-mediated oncogenes, such as HIF-1α and hexokinase, indicating that the direct transcriptional downregulation of p53 with PARP-1 upregulates cancer-related gene expression and phenotypes [60,61,62]. In other words, the mechanism of transcriptional regulation by PARP-1 in cancer cells can be considered as an important function that affects chromatin remodeling, regulation of tumor suppressor or oncogene expression, metastasis, and cancer cell survival (Figure 2). In particular, the p53 gene undergoes deletion in almost 90% of patients with pancreatic cancer, and approximately 60–70% of patients have point mutations that inactivate the remaining gene [63]. Hence, it is also an important research area to focus on the transcriptional regulation by PARP-1 in pancreatic cancer cells.

## 4. Clinical Study: PARP-1 Inhibitor Application in Patients with Pancreatic Cancer

Mutations involved in dsDNA break repair genes are known to respond to platinum-based chemotherapeutic agents, whereas mutations in homologous recombination (HR) genes follow the PARP-1 pathway, which is involved in repairing single-stranded DNA breaks as a rescue mechanism [64]. Furthermore, chemotherapy resistance is associated with an alteration in the tumor microenvironment or mechanisms of genetic changes, and it is particularly essential for causing genetic instability through genetic mutations involved in DNA damage repair [65]. Consequently, recent studies have confirmed that targeting PARP-1 has clinical benefits in BRCA-mutated patients with pancreatic cancer. As the application of PARP-1 inhibitor can induce a structural change in PARP-1 to pursue the continuous stabilization of PARP-1 and DNA combination, the PARP-1 inhibitor binds to the catalytic domain of PARP-1 to maintain a continuous active state of PARP-1 in cancer cells, and thus the catalytic cycle to return to the inactive state is finally interrupted, resulting in inducing PARP-1 dysfunction [66]. Another mechanism is to compete with NAD+ at the enzyme active site. PARP-1 inhibitors are largely based on benzamide or purine structures, and have the potential to inhibit other enzymes that use NAD+, including other members of the PARP family, mono-ADP-ribosyl-transferases [67]. Although PARP-1 inhibitors can seriously damage ssDNA break repair, dsDNA break repair plays a vital role in maintaining the integrity of the genetic material using HR as the compensation pathway. As described previously, BRCA-mutated cancer cells depend on non-HR and drive unique DNA repair systems that create specific genotypic and phenotypic features known as BRCAness [38,68]. Upstream molecular mutations regard the BRCAness as a major regulator related to mutation and deletion of PTEN, which may regulate RAD51 expression [69]. Downregulation of the form nuclear RAD51 foci has reduced the response to DNA damage by HR activity. The deficiencies in RAD51 expression can be translated into impaired dsDNA break repair [69,70]. Therefore, it can be inferred that BRCAness pancreatic cancer cells will not be able to repair HR deficiency; moreover, under the action of PARP inhibitors, the defective cells eventually succumb to synthetic lethality, and the sensitization of PARP-1 inhibitors will be beneficial in cancer treatment that is not dependent on the HR pathway.

To date, the following five PARP inhibitors have generated meaningful clinical results: olaparib, niraparib, veliparib, rucaparib, and talazoparib [71]. Olaparib is an oral PARP inhibitor that was first approved for the treatment of advanced ovarian cancer; however, today, it is applied to patients with recurrent pancreatic cancer carrying BRCA mutations [72]. In a prospective, multicenter, nonrandomized phase II study with olaparib oral monotherapy (400 mg twice per day) in BRCA-mutated patients with recurrent metastatic pancreatic cancer who received gemcitabine treatment, the tumor response rate was approximately 21.7% [73]. It is considered that the equivalence of efficacy has been confirmed when compared to existing regimens. In the first-line therapy, the disease response rate for gemcitabine and the nab-paclitaxel combination was approximately 23%, and that for FOLFIRINOX was approximately 31.6% [74]. The recent “pancreatic cancer olaparib ongoing (POLO)” study was conducted on BRCA-mutated patients with pancreatic cancer who did not progress following platinum-based chemotherapy that was randomly applied to 92 patients in a double-blind and placebo-controlled phase 3 clinical trial [75]. Comparative results showed that the median progression-free survival was 7.4 months in the olaparib group versus 3.8 months in the placebo group [75]. Niraparib is a highly selective inhibitor of PARP used for the treatment of patients with pancreatic cancer harboring deficiencies in HR, such as BRCAness. Niraparib is undergoing a phase 2 clinical trial to test its safety and effectiveness. However, the study is recruiting patients without interim reports [76,77]. In a single-arm, phase I clinical trial of gemcitabine, radiotherapy, and dose-escalated veliparib in patients with locally advanced pancreatic cancer, weekly gemcitabine treatment with daily intensity-modulated radiation therapy and dose-escalated veliparib was applied to 30 patients diagnosed with resectable pancreatic cancer [78]. That study confirmed that veliparib was safe and well-tolerated in combination therapy with gemcitabine and radiation therapy for patients with locally advanced pancreatic cancer [78]. A phase II study with rucaparib, which is also an oral PARP inhibitor, focused on the efficacy and safety of rucaparib in BRCA-mutated patients with measurable locally advanced, metastatic, pancreatic cancer [79]. The best response was a partial response in two patients, and one complete response was also confirmed. The disease control rate was 31.6% for all patients [79]. Talazoparib is a selective PARP inhibitor that is more potent than earlier generation PARP inhibitors. A multicenter, dose-escalation, phase I study was conducted to demonstrate the antitumor activity of talazoparib [80]. Of the 13 patients with pancreatic cancer, 4 demonstrated clinical benefits (two patients with partial response and the other two with stable disease) [80]. These clinical results well-supported the potential of these PARP inhibitors for further use in pancreatic cancer (Table 1). However, the toxicity that is accompanied by their positive outcomes is a concern that needs to be actively considered. PARP inhibitors can cause side effects with unacceptably high hematological toxicity and a sporadic association with acute myeloid leukemia in the long term. A combination of existing chemotherapeutics, such as gemcitabine, with veliparib or olaparib was associated with a marked increase of primarily hematological grade 3 toxicities according to the common terminology criteria for adverse events. Moreover, side effects were observed in 40% of patients with pancreatic cancer receiving olaparib alone, primarily including hematological toxicity, gastrointestinal side effects, fatigue, and lethargy (Table 1) [71,78,79,80,81]. Especially, when phase I study olaparib combination therapy was performed to determine the maximum tolerate dose of olaparib in combination with irinotecan and cisplatin, as well as the safety and tolerability of adding mitomycin, the trial results reported that olaparib in combination therapy showed significant toxicity in pancreatic cancer patients, such as drug-related myelodysplastic syndrome. It could not secure the noteworthy objective response rate and show an acceptable benefit profile to support further study [82]. Therefore, it is essential to consider the toxicity that may occur when PARP-1 inhibitors are used in combination with existing chemotherapeutics, and there needs to be potential solutions for optimizing therapy with sophisticated application therapies or by the development of new formulations.

## 5. Conclusions

The mechanistic roles of PARP-1 contributing to pancreatic cancer are being actively considered in various processes required for cancer cell survival, such as overcoming oxidative stress or genetic instability and regulation of mitosis and transcription. In particular, considering the genetic characteristics of the majority of pancreatic cancers using non-HR pathways and achieving significant clinical benefits by targeting PARP-1 are also of significant potential. However, the application of PARP-1 inhibitors targeting pancreatic cancer is still in its infancy, and there exists a need to remove several negative factors. It would be necessary to continue the discovery of various biomarkers that can be appropriately applied to the treatment of pancreatic cancer and the pathogenetic investigations to overcome the predicted toxicity or resistance.

## Figures and Tables

**Table 1:** Clinical trials of PARP-1 inhibitor for the treatment of pancreatic cancer.

Drugs	Trial ID	Outcomes	Toxicity
Olaparib	NCT02677038 NCT02511223	Objective response rate (PR: 6%; SD: 34%)	not available
Olaparib	NCT01078662	Tumor response rate (CR: 2%; PR: 32%; SD: 23%)	Above grade 3: nausea, fatigue, vomiting, anemia, abdominal pain, diarrhea, dyspnea
Veliparib	NCT00892736	Disease control rate (CR + PR: 23%; CR + PR + SD: 58%)	All grade: nausea, fatigue, and lymphopenia
Veliparib Gemcitabine	NCT01908478	Median OS (15 moths); tumor response rate (CR: 3%; SD: 93%)	Above grade 3: lymphopenia and anemia
Rucaparib	NCT03140670	Disease control rate (CR + PR + SD: 89.5%)	All grade: nausea, dysgeusia, fatigue
Rucaparib	NCT02042378	Disease control rate (PR + SD: 32%)	Above grade 3: nausea, anemia
Talazoparib	NCT01286987	Tumor response rate (PR: 15%; SD: 15%), median progression-free survival: 5.3 weeks	Above grade 3: fatigue, anemia, neutropenia, thrombocytopenia

## Data Availability

All collected data are included in this manuscript.

## Abbreviations

FOLFIRINOX	Fluorouracil, leucovorin, irinotecan, and oxaliplatin
RAS	rat sarcoma viral oncogenes homolog
PARP-1	Poly (adenosine diphosphate (ADP)-ribose) polymerase-1
PARylation	Poly-ADP-ribosylation
PAR	poly-ADP-ribose
NAD+	Nicotinamide adenine dinucleotide
ROS	Reactive oxygen species
NRF 2	Nuclear factor erythroid-related factor 2
HIF1-α	Hypoxia-inducing factor 1-alpha
AKT	Protein kinase B
ERK	Extracellular signal-regulated kinase
MKP-1	Mitogen-activated protein kinase phosphatase 1
MAPK	Mitogen-activated protein kinase
BRCA	Breast cancer susceptibility gene
ATM	Ataxia-telangiectasia mutated
ssDNA	Single-stranded DNA
dsDNA	Double-stranded DNA
